# Enhancing chemical reaction search through contrastive representation learning and human-in-the-loop

**DOI:** 10.1186/s13321-025-00987-5

**Published:** 2025-04-10

**Authors:** Youngchun Kwon, Hyunjeong Jeon, Joonhyuk Choi, Youn-Suk Choi, Seokho Kang

**Affiliations:** 1 Samsung Advanced Institute of Technology, Samsung Electronics Co. Ltd., 130 Samsung-ro, Yeongtong-gu, Suwon, Republic of Korea; 2 Department of Industrial Engineering, Sungkyunkwan University, 2066 Seobu-ro, Jangan-gu, Suwon, Republic of Korea

**Keywords:** Chemical reaction search, Contrastive learning, Human-in-the-loop, Graph neural network

## Abstract

In synthesis planning, identifying and optimizing chemical reactions are important for the successful design of synthetic pathways to target substances. Chemical reaction databases assist chemists in gaining insights into this process. Traditionally, searching for relevant records from a reaction database has relied on the manual formulation of queries by chemists based on their search purposes, which is challenging without explicit knowledge of what they are searching for. In this study, we propose an intelligent chemical reaction search system that simplifies the process of enhancing the search results. When a user submits a query, a list of relevant records is retrieved from the reaction database. Users can express their preferences and requirements by providing binary ratings for the individual retrieved records. The search results are refined based on the user feedback. To implement this system effectively, we incorporate and adapt contrastive representation learning, dimensionality reduction, and human-in-the-loop techniques. Contrastive learning is used to train a representation model that embeds records in the reaction database as numerical vectors suitable for chemical reaction searches. Dimensionality reduction is applied to compress these vectors, thereby enhancing the search efficiency. Human-in-the-loop is integrated to iteratively update the representation model by reflecting user feedback. Through experimental investigations, we demonstrate that the proposed method effectively improves the chemical reaction search towards better alignment with user preferences and requirements.

**Scientific contribution** This study seeks to enhance the search functionality of chemical reaction databases by drawing inspiration from recommender systems. The proposed method simplifies the search process, offering an alternative to the complexity of formulating explicit query rules. We believe that the proposed method can assist users in efficiently discovering records relevant to target reactions, especially when they encounter difficulties in crafting detailed queries due to limited knowledge.

## Introduction

A chemical reaction is a process in which substances, referred to as *reactants*, undergo chemical transformations to produce specific substances, referred to as *products*, under certain reaction conditions including the chemical context (e.g., catalysts, ligands, bases, solvents) and operating conditions (e.g., temperature and pressure). Identifying and optimizing chemical reactions are crucial for developing new functional materials [1]. To facilitate this, chemists leverage chemical reaction databases as invaluable resources to gain insights into the synthetic pathways towards the target substances [2–5]. These databases contain detailed records of chemical reactions that have been experimentally validated and published in the chemistry literature, providing essential information for replicating and refining reactions. Representative examples of these databases include Reaxys [6], SciFinder [7], Open Reaction Database (ORD) [8], and United States Patent and Trademark Office (USPTO) [9].

A chemical reaction search system assists chemists in obtaining relevant records from a chemical reaction database based on their specific interests. When a user submits a query specifying their search criteria, the system retrieves the most relevant records from the database. Research on chemical reaction search has focused on evaluating the similarity or relevance between a query and individual reaction records [10–12]. Existing databases also offer their own search engines based on straightforward search strategies [6–8]. These strategies include exact matching, similarity matching, and substructure matching. Exact matching retrieves reaction records in which at least one molecule exactly matches the queried molecule. Similarity matching retrieves records containing molecules whose similarity to the query exceeds a certain threshold. Substructure matching the retrieves records containing molecules that include the query as substructures. In addition, search constraints can be imposed to filter the retrieved records.

There are many possible querying scenarios in practice, each with different types of queries depending on the search objective [13]. The most typical scenarios of the search procedures are as follows. First, chemists query a target product of a reaction so that records producing this substance are retrieved. They then examine the reactants used in the records to determine possible synthetic paths for this product. Second, chemists query a target product along with its corresponding reactants to retrieve reaction records with exactly matching or similar substances. They then review the reaction conditions and reported yields to determine suitable conditions for a specific reaction. Third, chemists query all reaction information, including the product, reactants, reagents, and other required reaction conditions and measurements, to determine whether a chemical reaction with specific or similar conditions has been previously investigated.

**Fig. 1:**
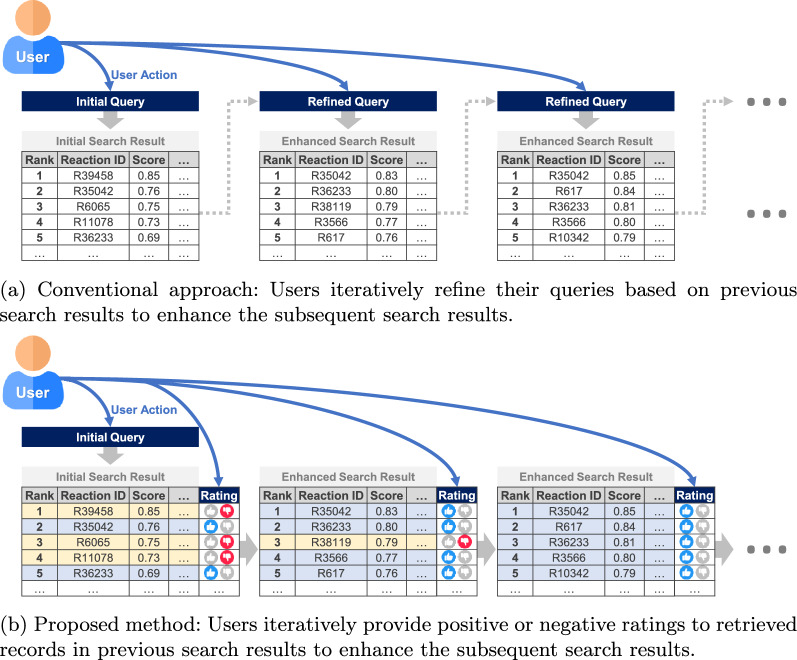
Schematic of chemical reaction search procedure

To customize the search results for search purposes, these three procedures are often performed sequentially and repeatedly until the desired reaction records are obtained. Based on previous search attempts, chemists derive their preferences and requirements in the form of explicit rules and manually incorporate these rules into their new queries as additional search constraints, such as blacklisting/whitelisting certain substructures or functional groups, as illustrated in Fig. 1a. If chemists have clear knowledge to identify the chemical reactions they are looking for, they can easily derive specific rules to retrieve more relevant records. Incrementally imposing and refining the constraints in the search queries allows users to retrieve what they want more effectively. However, if their knowledge of the target reaction for which they are searching is vague and limited, the rule-based approach may involve repetitive experimentation with various search queries on a trial-and-error basis to identify relevant records due to the difficulty of deriving explicit rules. While user feedback on retrieved records for a given query can be valuable for enhancing chemical reaction search, existing search engines in widely used reaction databases, such as Reaxys, SciFinder, and ORD, as well as existing studies on chemical reaction search, do not offer an automated way for users to incorporate their evaluations of retrieved records into the search results.

In this study, we aim to simplify the search process in the chemical reaction search system by allowing users to reflect their search preferences and requirements implicitly, rather than requiring the derivation of explicit rules in their queries. Inspired by how recommender systems work [14], we propose allowing users to provide binary ratings–positive or negative–for individual retrieved records in the search results, which can be regarded as implicit expressions of their search preferences and requirements. Subsequently, the search results are updated based on this feedback, as illustrated in Fig. 1b. To achieve this goal, we leverage contrastive representation learning, dimensionality reduction, and human-in-the-loop techniques. Contrastive learning is used to train a representation model that embeds reaction records as numeric vectors, thereby enabling the similarity between the user queries and reaction records to be measured through distance computations on the vector representations. Dimensionality reduction is applied to compress the vector representations to improve the efficiency of the distance computations. Human-in-the-loop is integrated to continuously update the representation model by incorporating user feedback on the retrieved records, thereby improving the subsequent search results.

## Method

### Problem definition

A reaction database, which consists of numerous reaction records, is used as the source for the chemical reaction search. Each reaction record consists of the meta information (e.g., the reaction ID and URL of the reference), products and reactants involved in the reaction, and reported reaction conditions (e.g., reagents, temperature, pressure, and reaction time), along with the corresponding reaction measurements (e.g., yield and conversion). It should be noted that each record may be associated with more than one set of reaction conditions. Although each record may contain multiple products, we limit our data to single-product reactions. In this context, any record with more than one product is decomposed into multiple records, each associated with a different single product while retaining the same reactants. For example, an original reaction record with three products is decomposed into three single-product reaction records.

We mathematically formulate the chemical reaction search as follows. Given a reaction database, each reaction record is embedded into a numeric vector $\textbf{x}_i$. Thus, we have the embedding vectors of *N* records from a reaction database in the form of $\mathcal {X} = \{ \textbf{x}_1, \ldots , \textbf{x}_N \}$. Once a query is made by a user, its embedding vector $\textbf{x}_*$ is obtained. We then search for the records whose embedding vectors are closest in distance to $\textbf{x}_*$.

The search performance, including the accuracy and efficiency, is highly dependent on how the query and reaction records are embedded into vectors, how the search algorithm is designed, and how user feedback is incorporated to enhance the search results. The details of how the proposed chemical reaction search system addresses these aspects are described in the following subsections.

### Representation model

The purpose of the representation model is to embed each reaction record into numeric vectors of a fixed size, enabling the similarity between chemical reactions to be evaluated using a readily calculable distance metric like Euclidean distance. To be used as input to the model, we transform each reaction record into a tuple $(\mathcal {G}^P, \mathcal {G}^R, \mathcal {G}^A)$, where $\mathcal {G}^P$, $\mathcal {G}^R$, and $\mathcal {G}^A$ denote the graph representations of the product, reactants, and reagents, respectively. In the graph representation, the nodes and edges are associated with heavy atoms and their bonds, respectively [15]. Hydrogen atoms are treated implicitly as node features, implying that there are as many nodes as heavy atoms in the corresponding molecule. The node features include the atomic number, formal charge, degree, hybridization, number of hydrogen atoms, chirality (CW or CCW), whether it is aromatic, whether it is in a ring, and the associated ring sizes. The edge features include the bond type, bond direction (end-upright or end-downright), stereochemistry (E or Z), whether it is in a ring, and whether it is conjugated. It should be noted that multiple reactants and reagents may exist in a record, in which the corresponding graph representation $\mathcal {G}^R$ or $\mathcal {G}^A$ consists of multiple subgraphs that are not interconnected.

**Fig. 2:**
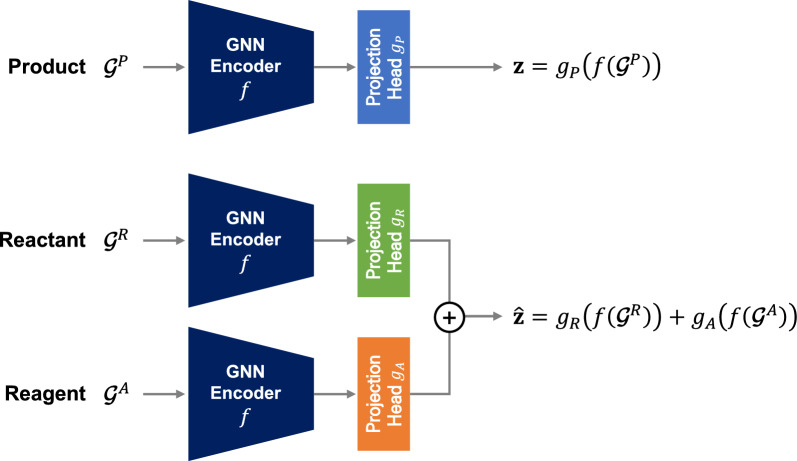
Architecture of the representation model

Figure 2 illustrates the architecture of the representation model, which consists of a graph neural network (GNN) encoder *f* and three projection heads $g_P$, $g_R$, and $g_A$ for the product, reactants, and reagents, respectively. The GNN encoder *f* maps an input graph to a vector representation. Since the input graph may contain more than one molecule, we employ sum pooling as the readout function in the GNN encoder *f*. This ensures that stoichiometry is accounted for by preserving the quantitative aspects of the input in the output vector representation [16]. The GNN encoder *f* is shared across the processing of $\mathcal {G}^P$, $\mathcal {G}^R$, and $\mathcal {G}^A$. Each projection head, $g_P$, $g_R$, or $g_A$, maps the output of *f* to its projection. Given an instance $(\mathcal {G}^P, \mathcal {G}^R, \mathcal {G}^A)$, the product $\mathcal {G}^P$ is processed by the GNN encoder *f* and then further processed by the projection head $g_P$ to obtain a projection, which we refer to as the *target vector* $\textbf{z}$, as follows:

1 $$\begin{aligned} \textbf{z} = g_P(f(\mathcal {G}^P)) \in \mathbb {R}^p. \end{aligned}$$

Similarly, the reactants $g_R$ and reagents $g_A$ are also processed by the shared GNN encoder *f* and their respective projection heads $g_R$, and $g_A$. The summation of the two vectors, $g_R(f(\mathcal {G}^R))$ and $g_A(f(\mathcal {G}^A))$, which we refer to as the *prediction vector* $\hat{\textbf{z}}$, is obtained as follows:

2 $$\begin{aligned} \hat{\textbf{z}} = g_R(f(\mathcal {G}^R))+g_A(f(\mathcal {G}^A)) \in \mathbb {R}^p. \end{aligned}$$

To approximately zero-center the target and prediction vectors over the database, no bias terms were used in the final layers of the projection heads.

It should be noted that reagents may be missing in some reaction records within the database. If all reagents $\mathcal {G}^A$, including catalysts, ligands, bases, solvents, are missing in a record, then the reagent embedding $g_A(f(\mathcal {G}^A))$ becomes a zero vector, exerting no effect on the prediction vector $\hat{\textbf{z}}$. If some of the reagents are present, this is reflected in the prediction vector $\hat{\textbf{z}}$ by the deviation of the reagent embedding $g_A(f(\mathcal {G}^A))$ from zero. To further enhance reagent-related searches, it would be beneficial to complete the missing reagents in reaction records within the database using reaction condition prediction methods as missing imputers [4, 17–19].

### Representation learning on chemical reactions

Research on representing chemical reactions in vectors ranges from handcrafted reaction fingerprints [20–22] to data-driven representation learning using neural networks [23–26]. While reaction fingerprints are simple and efficient, representation learning offers a highly expressive means of capturing detailed and nuanced information regarding molecular structures in reactions, with the adaptability of incorporating user preferences into the learning objective. Representation learning works by building a model that embeds the original representation into a vector. Schwaller et al. [23] trained a BERT model on ReactionSMILES strings using masked language modeling. More recently, Wang et al. [24], Wen et al. [25], and Xie et al. [26] trained GNNs on the graph representations of molecules in reactions by leveraging contrastive learning.

Contrastive learning aims to learn representations by distinguishing between similar and dissimilar instances, bringing positive pairs closer and pushing negative pairs further apart in the embedding space [27, 28]. Wang et al. [24] built a representation model that separately embeds products and reactants using a learning objective that treats products and reactants from the same reaction as positive pairs and those from different reactions as negative pairs. Wen et al. [25] built a representation model that provides reaction-level embeddings. Using a reaction data augmentation technique, the learning objective defines positive pairs as the augmented views of the same reaction and negative pairs as those from different reactions. Xie et al. [26] decomposed each reaction into multiple reactant-template-product triplets and built a representation model with the learning objective of aligning the sum of the reactant and template embeddings with the product embedding for each reaction.

Similar to the latter studies [24–26], we train our representation model based on contrastive learning with the objective that the target and prediction vectors for the same reaction are close to one another, whereas the vectors for different reactions are far apart. Typically, contrastive learning focuses on training a representation model to capture generally meaningful features for pretraining purposes, enabling the model to be fine-tuned effectively for various downstream tasks [27–29]. In contrast, we focus on training a representation model to create an embedding space in which the similarity between instances can be measured directly using a specific distance metric, aligning with the purpose of metric learning [30]. In further relation to existing studies, our representation model separately embeds products and reactants [24, 26]. The way of defining positive and negative pairs for contrastive learning is similar to that used by Wang et al. [24]’s study. In addition, we do not use data augmentation techniques, because perturbing the nodes and edges in molecular graphs can potentially disrupt the intrinsic properties of the molecules involved in the reactions [31–33].

The training dataset containing *N* reaction records has the form of $\mathcal {D}=\{(\mathcal {G}^P_i, \mathcal {G}^R_i, \mathcal {G}^A_i)\}_{i=1}^N$. In each training iteration, given a minibatch $\mathcal {S}=\{(\mathcal {G}^P_i, \mathcal {G}^R_i, \mathcal {G}^A_i)\}_{i=1}^M$ sampled from $\mathcal {D}$, we generate the target vector $\textbf{z}_i$ and prediction vector $\hat{\textbf{z}}_i$ for each reaction, resulting in a total of 2*M* vectors $\textbf{z}_1,\ldots ,\textbf{z}_M,\hat{\textbf{z}}_1,\ldots ,\hat{\textbf{z}}_M$. We use the vector pair $\textbf{z}_i$ and $\hat{\textbf{z}}_i$ for every reaction as the positive pair, while all other pairs are used as the negative pairs for contrastive learning. This leads to *M* positive pairs and $2M(M-1)$ negative pairs. For this purpose, we employ a modified version of the normalized temperature-scaled cross entropy (NT-Xent) loss [27], where we replace the cosine similarity with the negative squared Euclidean distance. The contrastive loss function $l_c$ is expressed as:

3 $$\begin{aligned} l_c(i,j) = -\log \frac{ \exp ({- d^2( \textbf{z}_i, \textbf{z}_j) / \tau }) }{\sum _{k=1}^{2M} {\textbf{1}(i\ne k) \exp (-d^2( \textbf{z}_i, \textbf{z}_k) / \tau }) }, \end{aligned}$$

where $d^2$ is the squared Euclidean distance and $\tau$ is the temperature hyperparameter. Minimizing $l_c(i,j)$ implies that the distance between $\textbf{z}_i$ and $\textbf{z}_j$ is reduced relative to the distances between $\textbf{z}_i$ and $\textbf{z}_k$ for all $k\ne j$.

The final learning objective, computed on the minibatch $\mathcal {S}$, is derived as:

4 $$\begin{aligned} \mathcal {J} = \frac{1}{2M} \sum _{i=1}^M {[l_c(i, M+i)+l_c(M+i, i)]}, \end{aligned}$$

where we let $\textbf{z}_{M+i} = \hat{\textbf{z}}_i$ for notational simplicity. By minimizing $\mathcal {J}$, the parameters of the representation model are updated such that the representations of each positive pair are close and those of each negative pair are far apart.

### Dimensionality reduction

Once the representation model is trained, the target and prediction vectors, $\textbf{z}_i$ and $\hat{\textbf{z}}_i$, can be obtained for each *i*-th record in the original dataset $\mathcal {D}$. Given a query, the chemical reaction search can be implemented by retrieving the records with low distances in their vector representations, necessitating the distance calculations among vectors between the query and records. The issue is that the cost of computation and data storage increases with the dimensionality *p*.

To improve search efficiency, we apply principal component analysis (PCA) to reduce the dimensionality of the target and prediction vectors to $q \ll p$. We construct a $2N\times p$ matrix $\textbf{Z}=[\textbf{z}_1;\cdots ;\textbf{z}_N;\hat{\textbf{z}}_1;\cdots ;\hat{\textbf{z}}_N]$. We then apply a low-rank approximation of singular value decomposition (SVD) to the matrix to obtain *q* principal directions, leading to the factorization of the three matrices as follows:

5 $$\begin{aligned} \textbf{Z}=[\textbf{z}_1;\cdots ;\textbf{z}_N;\hat{\textbf{z}}_1;\cdots ;\hat{\textbf{z}}_N]=\textbf{U} \textbf{S} \textbf{V}^T, \end{aligned}$$

where $\textbf{U}$ is a $2N\times q$ matrix, $\textbf{S}$ is a $q \times q$ diagonal matrix, and $\textbf{V}$ is a $p \times q$ matrix. Each column in $\textbf{V}$ represents a principal direction.

Using the *q* principal directions in $\textbf{V}$, we project the original vectors $\textbf{z}_i$ and $\hat{\textbf{z}}_i$ onto the *q* principal components as follows:

6 $$\begin{aligned} \begin{aligned} \textbf{z}'_i =&\textbf{z}_i \textbf{V} \in \mathbb {R}^q;\\ \hat{\textbf{z}}'_i =&\hat{\textbf{z}}_i \textbf{V} \in \mathbb {R}^q, \end{aligned} \end{aligned}$$

resulting in projected vectors with a reduced dimensionality *q*.

Once Euclidean distance is used to measure pairwise distances between vectors, the distance calculations can be well-approximated in the lower-dimensional space projected by PCA. The following equation shows how the squared Euclidean distance between two projected vectors $d^2(\textbf{z}'_i,\textbf{z}'_j)=(\textbf{z}'_i-\textbf{z}'_j)^T(\textbf{z}'_i-\textbf{z}'_j)$ relates to that of the original vectors $d^2(\textbf{z}_i,\textbf{z}_j)=(\textbf{z}_i-\textbf{z}_j)^T(\textbf{z}_i-\textbf{z}_j)$:

7 $$\begin{aligned} (\textbf{z}'_i-\textbf{z}'_j)^T(\textbf{z}'_i-\textbf{z}'_j) =(\textbf{z}_i \textbf{V}-\textbf{z}_j \textbf{V})^T(\textbf{z}_i \textbf{V} -\textbf{z}_j \textbf{V}) = (\textbf{z}_i-\textbf{z}_j)^T(\textbf{z}_i-\textbf{z}_j) \textbf{V}^T \textbf{V}. \end{aligned}$$

If the number of principal directions *q* is set equal to *p*, then the matrix $\textbf{V}^T \textbf{V}$ becomes an identity matrix owing to the orthonormality of the principal components, and therefore, the two distances are identical. By setting *q* to be sufficiently large such that the explained variance ratio is close to 1, $\textbf{V}^T \textbf{V}$ remains close to an identity matrix, ensuring that the Euclidean distance computed in the projected space closely approximates that in the original space.

A larger dimensionality *q* enhances approximation accuracy but increases computational costs, whereas a smaller *q* reduces costs at the expense of accuracy. To balance this trade-off in approximate Euclidean distance calculations, the dimensionality *q* can be selected such that the explained variance ratio of the principal components meets a certain threshold (e.g., 95%). The use of the projected vectors, $\textbf{z}'_i$ and $\hat{\textbf{z}}'_i$, reduces the computational and data storage costs required for reaction search by a factor of approximately *q*/*p*.

### Search algorithm

**Fig. 3:**
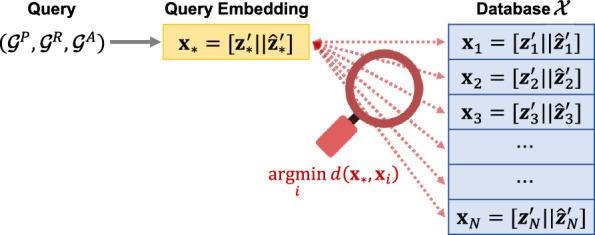
Schematic of chemical reaction search

For a chemical reaction search, a user provides a query specifying the reaction context $(\mathcal {G}^P_*, \mathcal {G}^R_*, \mathcal {G}^A_*)$ and ranges of reaction conditions and measurements. At least one of the product $\mathcal {G}^P_*$ or reactant $\mathcal {G}^R_*$ must be provided in the query. How the query embedding is obtained depends on the search strategy the user intends to use. In the case of exact or similarity matching, where the query consists of complete molecules, we simply embed the query using the representation model. For substructure matching, where the query consists of substructures that must be included in the corresponding reaction context, we first select reaction records from the database that explicitly contain these queried substructures. The query embedding is then computed as the mean of the embeddings of these selected records. After reducing the dimensionality, we obtain the query vector $\textbf{x}_* = [\textbf{z}'_* \Vert \hat{\textbf{z}}'_*]$, where $\Vert$ is the concatenation operator. If the reactant is not provided in the query, $\textbf{z}'_*$ is used instead of $\hat{\textbf{z}}'_*$ (i.e., $\textbf{x}_* = [\textbf{z}'_* \Vert \textbf{z}'_*]$). Similarly, if the product is not provided, $\hat{\textbf{z}}'_*$ is used instead of $\textbf{z}'_*$ (i.e., $\textbf{x}_* = [\hat{\textbf{z}}'_* \Vert \hat{\textbf{z}}'_*]$).

The search process is illustrated in Fig. 3. Among the reaction records in the database that satisfy the user’s specifications, we search the records that best match the query from the database $\mathcal {X}=\{\textbf{x}_1,\ldots ,\textbf{x}_N\}$, where $\textbf{x}_i=[\textbf{z}'_i \Vert \hat{\textbf{z}}'_i]$ is the embedding for the *i*-th record. If the specified ranges of any attributes are provided, records outside these ranges are filtered out. Subsequently, the chemical reaction search is formulated as the retrieval of records with the lowest distances. The distance between the query and each *i*-th record is calculated as $d(\textbf{x}_*, \textbf{x}_i)$. The top-*K* retrieved records $\textbf{x}_{*}^{(1)},\ldots ,\textbf{x}_{*}^{(K)}$, in ascending order of distance, are provided to the user.

### Model updating based on user feedback

For each query $\textbf{x}_*$, the search result contains the top-*K* retrieved records $\textbf{x}_*^{(1)},\ldots ,\textbf{x}_*^{(K)}$. Based on the search preferences and requirements, users can rate the relevance of each retrieved record to the query as positive (+1), negative (-1), or neutral/no answer (0). We denote the user rating for each record $\textbf{x}_*^{(i)}$ by $r_*^{(i)} \in \{-1, 0, +1\}$.

We introduce the human-in-the-loop learning procedure [34] to further enhance the search results. The goal of human-in-the-loop is to incorporate human expertise and feedback into the learning process of a model to continuously improve its performance. As user feedback is provided in the form of binary ratings for the retrieved records for each query, we iteratively update the representation model to reflect these ratings in the previous search results. This allows users to customize their subsequent search results to increase satisfaction.

After updating the representation model, records with positive ratings should be ranked higher, whereas those with negative ratings should be ranked lower. If $r_{*}^{(i)} > r_{*}^{(j)}$, then $d(\textbf{x}_*,\textbf{x}_{*}^{(i)}) < d(\textbf{x}_*,\textbf{x}_{*}^{(j)})$. Conversely, if $r_{*}^{(i)} < r_{*}^{(j)}$, then $d(\textbf{x}_*,\textbf{x}_{*}^{(i)}) > d(\textbf{x}_*,\textbf{x}_{*}^{(j)})$. To achieve this for all pairs of *K* retrieved records for a query, we use the margin ranking loss function $l_r$ defined as follows:

8 $$\begin{aligned} \begin{aligned} l_r(\textbf{x}_*)&= \frac{2}{K(K-1)}\sum _{i=1}^{K-1} \sum _{j=i+1}^{K} \\&\max \left( 0, (r_{*}^{(i)}-r_{*}^{(j)}) \cdot (d(\textbf{x}_*,\textbf{x}_{*}^{(i)})-d(\textbf{x}_*,\textbf{x}_{*}^{(j)})) + |r_{*}^{(i)}-r_{*}^{(j)}|\cdot \delta \right) , \end{aligned} \end{aligned}$$

where $\delta$ is a margin hyperparameter. Minimizing $l_r(\textbf{x}_*)$ encourages the distances between the query and positively rated records to be relatively smaller than the distances between the query and negatively rated records by a margin in the embedding space.

Given a query set $\mathcal {Q}$ containing recent user queries and the ratings for retrieved records, the learning objective $\tilde{\mathcal {J}}$ for updating the representation model is given by:

9 $$\begin{aligned} \tilde{\mathcal {J}} = \mathcal {J} + \lambda \cdot \frac{1}{|\mathcal {Q}|} \sum _{\textbf{x}_* \in \mathcal {Q}} l_r(\textbf{x}_*) , \end{aligned}$$

where the first term $\mathcal {J}$ is the original learning objective used for contrastive representation learning, the second term is the average of the ranking losses for queries in $\mathcal {Q}$, and $\lambda$ is a trade-off hyperparameter. The first term is used to maintain overall representation quality and stabilize fine-tuning, which can be especially helpful when user feedback is inconsistent or contradictory across different queries and records. The second term ensures that the representation model reflects the user ratings. We fine-tune the representation model by minimizing the objective $\tilde{\mathcal {J}}$. After fine-tuning, we update the embedding vectors of the reaction database using the improved representation model, thereby enhancing the subsequent search results for user queries.

## Results

### Data

We used the USPTO-479k dataset [35], which consists of records of 478,612 chemical reactions, for evaluation purposes. Each reaction comprised up to five reactants and exactly one product. The dataset was originally divided into training, validation, and test sets with 408,673, 29,973, and 39,966 reactions, respectively. These splits were retained without modification.

It should be noted that the reaction records in the USPTO-479k dataset contain only products and reactants, with no information on the reagents. Therefore, reagent embedding was not used in this implementation.

### Implementation details

For the representation model, we configured the architecture of the GNN encoder *f* as a graph isomorphism network (GIN) [36, 37]. GIN had empirically demonstrated high expressive power on graph-structured data like molecular graphs [36]. Specifically, we adopted a variant of GIN that incorporated edge features [37]. The GNN encoder *f* employed a five-layer GIN architecture, with each layer having a dimensionality of 300, following the default setting in [37]. We used sum pooling as the readout function to account for stoichiometry [16]. Each projection head, $g_P$, $g_R$, and $g_A$, consisted of two fully-connected layers, each with 512 dimensions, with ReLU activation applied in the first layer. The dimensionality *p* was set to 512.

For representation learning on chemical reactions, we trained the representation model using the Adam optimizer with a learning rate of $10^{-4}$, minibatch size *M* of 4096, and weight decay of $10^{-8}$. The temperature hyperparameter $\tau$ was set to 100. The training was terminated if the number of epochs reached 200 or the validation loss did not decrease for 20 consecutive epochs.

**Fig. 4:**
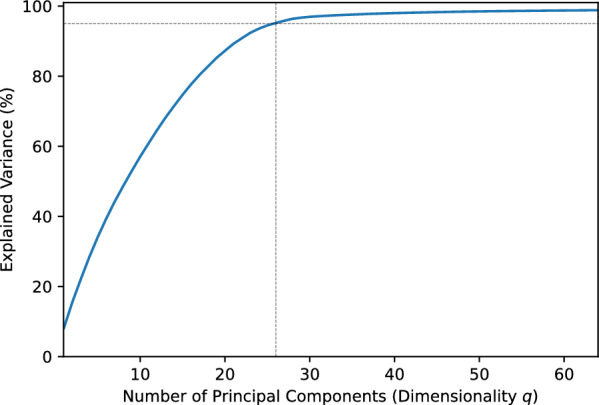
Explained variance according to the number of principal components

For dimensionality reduction of the representations, Fig. 4 plots the explained variance ratio against the number of principal components obtained by applying PCA. We set the reduced dimensionality *q* to 26, which corresponded to an explained variance of 95%. Accordingly, the dimensionality of the target and prediction vectors was reduced from 512 to 26, resulting in a compression rate of 94.9%.

For the search algorithm, the distance measure *d* was set as the Euclidean distance to be aligned with the loss function used in the contrastive representation learning.

For model updating based on user feedback, we fine-tuned the representation model for 100 iterations using the stochastic gradient descent (SGD) optimizer with a learning rate of $10^{-4}$, momentum of 0.9, and weight decay of $10^{-8}$. Batch normalization in the GNN encoder *f* was switched off. The minibatch *S* was randomly sampled from the training dataset $\mathcal {D}$ when calculating the learning objective $\tilde{\mathcal {J}}$ at each iteration. The hyperparameters $\delta$ and $\lambda$ were set to 100 and 0.01, respectively.

The experiments were conducted on a single NVIDIA RTX 3090 GPU with 24GB of memory. In contrastive representation learning, GPU memory availability limits the maximum minibatch size we can use, an increase in which generally improves contrastive learning performance [24, 25, 27].

### Reaction product prediction

We evaluated the quality of the embedding vectors obtained by the representation model using the reaction product prediction task, following the work of Wang et al. [24] and Xie et al. [26]. The reaction product prediction task involves determining whether the ground-truth product can be retrieved from a pool of candidate products when certain reactants are provided solely as a search query.

To formulate the reaction product prediction task, we used the 39,458 unique products in the test set as the candidate pool. Given the reactants in each *i*-th reaction record in the test set, we calculated the distance between the reactant embedding $\hat{\textbf{z}}_i$ and each product embedding $\textbf{z}_j$ in the candidate pool, i.e., $d(\hat{\textbf{z}}_i, \textbf{z}_j )$. Then, by ranking all candidate products in the order of distance, we determined the ranking of the ground-truth product within the candidate pool.

We evaluated two versions of the proposed method based on whether dimensionality reduction was applied. For the version without dimensionality reduction, we varied the dimensionality *p* among 512, 128, and 32 to assess its effect on performance. For the version with dimensionality reduction using PCA, we fixed $p=512$ and varied the reduced dimensionality *q* among 77, 41, and 26, corresponding to explained variance ratios of 99%, 98%, and 95%, respectively.

For the baseline methods, we compared Mol2vec [38], MolBERT [39], MolR [24], and ReaKE [26], all of which were trained or fine-tuned using USPTO-479k. The results of these baselines were taken from the work of Wang et al. [24] and Xie et al. [26].

The performance of each method was evaluated by calculating the following measures on the test set: the mean reciprocal rank (MRR), mean rank (MR), and hit ratios at the top-1, -3, -5, and -10 retrieved records (Hit@1, Hit@3, Hit@5, and Hit@10).

Table 1 compares the reaction product prediction performance of the baseline and proposed methods. The results show that the proposed method achieved the best performance across all performance measures, indicating that it effectively learned representations where the embedding of the reactants in a reaction was close to that of the product in the same reaction. For the proposed method, when dimensionality reduction was not applied, a higher dimensionality *p* led to better performance. When the original dimensionality *p* was set to a high value (*p*=512) and PCA was applied for dimensionality reduction, the performance remained nearly unchanged compared to the case without dimensionality reduction. This suggests that dimensionality reduction can make the prediction process much faster and more efficient without compromising accuracy. Notably, the proposed method with $p=512$ and $q=26$, the default setting, achieved a Hit@1 of 0.966, meaning that the highest-ranked candidate product exactly matched the ground-truth product in 96.6% of the test reactions.

**Table 1:** Comparison of reaction product prediction performance

Method	MRR$\uparrow$	MR$\downarrow$	Hit@1$\uparrow$	Hit@3$\uparrow$	Hit@5$\uparrow$	Hit@10$\uparrow$
Mol2vec [38]	0.688	417.6	0.620	0.734	0.767	0.806
MolBERT [39]	0.776	459.6	0.708	0.827	0.859	0.891
MolR [24]	0.918	27.4	0.882	0.949	0.960	0.970
ReaKE [26]	0.967	2.9	0.950	0.982	0.987	0.992
Proposed (w/o Dim. Reduction)						
$p=512$	**0.981**	**1.3**	**0.967**	**0.995**	**0.997**	**0.998**
$p=128$	0.980	1.4	0.965	**0.995**	**0.997**	**0.998**
$p=32$	0.977	1.6	0.960	0.994	0.996	**0.998**
Proposed (w/ Dim. Reduction)						
$p=512, q=77 \text {(99\% Explained Var.)}$	**0.981**	**1.3**	**0.967**	**0.995**	**0.997**	**0.998**
$p=512, q=41 \text {(98\% Explained Var.)}$	**0.981**	**1.3**	0.966	**0.995**	**0.997**	**0.998**
$p=512, q=26 \text {(95\% Explained Var.)}$	0.980	1.4	0.966	**0.995**	**0.997**	**0.998**

Bold values indicate the best result for each performance measure

### Chemical reaction search with user feedback

We evaluated the effectiveness of the proposed method in enhancing search results for user queries by incorporating user feedback. We considered two query types for similarity matching: (1) the target product is specified only in a query; (2) both the target product and reactants are specified in a query. To compose the search queries for each query type, we randomly sampled 10 reaction records from the test set. The training and validation sets were used as the reaction database to be searched.

We simulated chemical reaction searches using user feedback based on specific user preference scenarios designed by human experts. In collaboration with three experienced experimental chemists from the Samsung Advanced Institute of Technology (SAIT), we identified general user preferences during reaction searches. Through discussions, we derived five typical user preference scenarios that reflect the common considerations of chemists when conducting searches. For each scenario, we designed a simplified condition for a positive rating. The five user preference scenarios and their conditions for positive ratings are listed in Table 2. User ratings for the individual retrieved records in the search results were assigned based on whether they satisfied the specified condition in the corresponding scenario. Each record received a positive rating (+ 1) if it met the condition and a negative rating (− 1) if it did not.

The evaluation procedure for each scenario was as follows. Given a set of queries, we retrieved 30 relevant records from the database for each query. Subsequently, these retrieved records were rated as positive or negative based on the specified user preference condition in the scenario. The representation model was then updated to reflect the user ratings, and the search results were refreshed by retrieving 30 relevant records per query again. This updating process was repeated three times.

Dimensionality reduction was applied to the embedding vectors to enhance the efficiency for the search process. Assuming a resource-constrained environment, we measured the retrieval speed using a single CPU core. Without dimensionality reduction, the average CPU time required to retrieve the search results was 0.64 s per query. After applying dimensionality reduction, this time decreased to 0.07 s per query, indicating a significant improvement in speed.

**Fig. 5:**
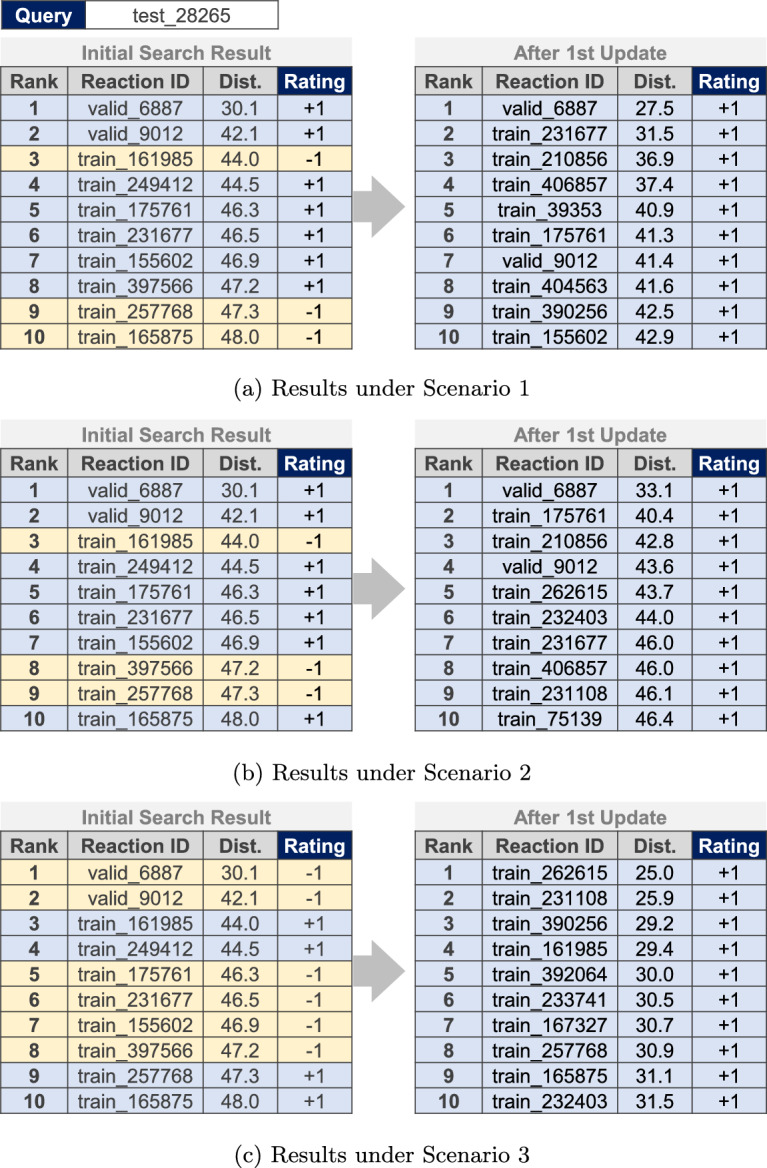
Examples of chemical reaction search results before and after the first updates for the same query under different user preference scenarios

Figure 5 shows examples of the top 10 retrieved reactions before and after the first updates for the same test query under three different user preference scenarios. For each scenario, different reaction records were positively rated according to the respective conditions specified in Table 2, leading to search result updates in different directions. Positively rated records were consistently retained in the search results, whereas negatively rated records were removed after the update. In addition, the newly retrieved records in the search results tended to meet the specified conditions, thereby improving the hit ratios.

**Fig. 6:**
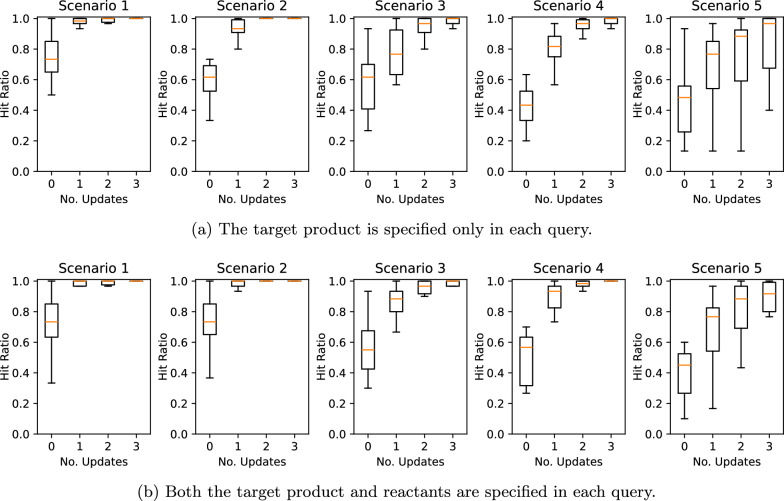
Chemical reaction search performance according to the number of updates across various query types and user preference scenarios

We evaluated the quality of the retrieved records for each query in terms of the hit ratio, the fraction of positively rated retrieved records. Figure 6 plots the average hit ratios across queries against the number of updates for all combinations of query types and user preference scenarios. The results show that the average hit ratio consistently improved with each update across all cases, suggesting that incorporating human feedback is effective in enhancing the search results according to user preferences. Notably, the most significant improvement occurred after the first update in all cases. When the specified condition for a positive rating became more complex, involving a mixture of preferences that better aligned with real-world situations, the performance improvement slowed.

**Table 2:** User preference scenarios

Scenario ID	Condition for positive rating
1	The product in the retrieved reaction contains the same halogen atoms as the product in the query.
2	The number of reactants in the retrieved reaction matches the number of reactants in the query (or is 2 if no reactants are specified in the query).
3	The maximum Tanimoto similarity between the product and any reactant in the retrieved reaction exceeds 0.5.
4	Both conditions in Scenarios 1 and 2 are satisfied in the retrieved reaction.
5	Both conditions in Scenarios 1 and 3 are satisfied in the retrieved reaction.

## Conclusion

In this study, we have presented an enhanced chemical reaction search system that automatically incorporates user feedback to improve the search results. It leverages contrastive representation learning and human-in-the-loop techniques to learn from both the reaction database and user input. In response to a query, users can provide a binary rating for each retrieved record. These ratings are then used to refine the search results by aligning them more closely with user expectations. Through experimental investigations, we found that the proposed method improved the search results by effectively integrating user feedback.

The quality of search results in chemical reaction searches has traditionally relied solely on the accuracy and concreteness of the search queries. However, manually curating and refining a query is challenging when users lack clear knowledge of the target reactions. The proposed method allows users to express their preferences and requirements through the binary ratings of retrieved records, thereby simplifying the search process compared to the complexity of deriving explicit rules for query formulation. We believe that the proposed method can help users to discover records relevant to the target reactions more efficiently, particularly when they struggle to formulate detailed queries owing to limited knowledge.

Although the proposed method can assist chemists in retrieving valuable reaction records from a reaction database, several practical issues require further investigation. We outline three potential research directions for future work to improve the usability and applicability of the proposed method. First, reflecting user feedback requires updating the representation model and vector embeddings, which is currently computationally expensive and time-consuming. Improving the efficiency is crucial to enable real-time updates that promptly reflect user feedback. Second, identifying commonalities among positively/negatively rated reaction records can provide users with valuable insights. Integrating systematic interpretations of these commonalities into the search system can help users to better understand and refine their knowledge of the target reactions. Third, extending the search system to include reagent recommendations by identifying commonly used reagents (e.g., catalysts, ligands, bases, and solvents) in positively rated reaction records would further enhance its utility.

## Data Availability

The source code used in this study, including running examples on the USPTO-479k dataset, is available online at https://github.com/seokhokang/reaction_search.
